# The protective role of resveratrol against high glucose‐induced oxidative stress and apoptosis in HepG2 cells

**DOI:** 10.1002/fsn3.4027

**Published:** 2024-02-16

**Authors:** Abegail Mukhethwa Tshivhase, Tandi Matsha, Shanel Raghubeer

**Affiliations:** ^1^ SAMRC/CPUT Cardiometabolic Health Research Unit, Department of Biomedical Sciences, Faculty of Health and Wellness Sciences Cape Peninsula University of Technology Bellville South Africa; ^2^ Sefako Makgatho Health Sciences University Ga‐Rankuwa South Africa

**Keywords:** apoptosis, high glucose, hyperglycemia, oxidative stress, resveratrol

## Abstract

High glucose concentrations result in oxidative stress, leading to damage of cellular constituents like DNA, proteins, and lipids, ultimately resulting in apoptosis. Resveratrol, a polyphenol phytoalexin, has been studied for its potential therapeutic effects on diabetes. This study investigated the influence of high glucose (HG) on HepG2 cells and assessed resveratrol's effect on high‐glucose‐induced oxidative stress and apoptosis. HepG2 cells were cultured for 48 and 72 h with high glucose (40 mM), low resveratrol (25 μM), high resveratrol (50 μM), high glucose + low resveratrol, and high glucose + high resveratrol. After exposure, oxidative and apoptosis‐related gene expression was evaluated using quantitative polymerase chain reaction (qPCR), and lactate dehydrogenase (LDH) release was measured using the supernatant. In HepG2 cells cultured with high glucose, all antioxidant enzymes (SOD, superoxide dismutase; GPx1, glutathione peroxidase 1; CAT, catalase; Nrf2, nuclear factor erythroid 2–related factor 2; and NQO1, NAD(P)H quinone oxidoreductase 1) were significantly reduced; however, when HepG2 cells were cultured with resveratrol (25 and 50 μM) and high glucose, the expression levels of all antioxidant enzymes were increased. The anti‐apoptotic gene (B‐cell lymphoma 2; *Bcl2*) and the DNA repair gene (Oxoguanine glycosylase‐1, *OGG1*) were significantly decreased following high glucose exposure to HepG2 cells. Surprisingly, the expression levels of *Bcl2* and *OGG1* were notably elevated after resveratrol treatment. Furthermore, high glucose levels increased the LHD release in HepG2 cells, whereas resveratrol treatment reduced the LDH release. Our results demonstrate that resveratrol provides protection against oxidative stress and apoptosis induced by high glucose in HepG2 cells. Hence, resveratrol shows potential as an effective approach to address the impaired antioxidant response resulting from elevated glucose levels commonly observed in diabetes and metabolic disorders.

## Graphical abstract

Our investigation into the effects of resveratrol on HepG2 cells demonstrates that resveratrol protects against high glucose‐induced oxidative stress and apoptosis in HepG2 cells. Therefore, resveratrol may serve as a promising strategy to combat antioxidant response dysfunction caused by high glucose levels characteristic of diabetes conditions and metabolic disorders.

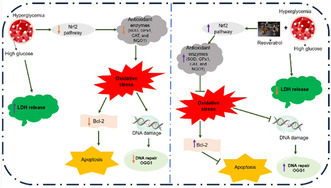

## 1 INTRODUCTION

Diabetes mellitus (DM) refers to a metabolic disorder marked by elevated levels of blood glucose and inadequate insulin production or action (Solis‐Herrera et al., 2018). The International Diabetes Federation (IDF) reported that the global prevalence of diabetes was 537 million in 2021, with projections indicating a rise to 643 million by 2030 and 745 million by 2045 (Sun et al., 2022). This has become an urgent public health threat due to its rising incidence. Hyperglycemia is accompanied by micro‐ and macrovascular complications and multi‐organ damage (Kapoor & Kakkar, 2012). Hence, it is severely essential for hyperglycemia to be treated effectively to inhibit these complications and improve the patient's outcome.

The liver serves as the main organ responsible for glucose metabolism and regulation (Dey & Chandrasekaran, 2009). Consequently, it is of considerable interest to investigate the impacts of hyperglycemia on liver cells cultured in vitro. The HepG2 cell line, derived from human hepatoma, has been widely employed in vitro to investigate hyperglycemia (Chandrasekaran et al., 2010). Moreover, HepG2 cells exhibit certain physiological characteristics akin to those of the human liver (Knowles et al., 1980). In hyperglycemia, high glucose levels can increase reactive oxygen species (ROS), overwhelming the antioxidant defense of the body and producing oxidative stress (Bhatti et al., 2022). Previous research has linked oxidative stress with the onset and progression of DM (Subramaniyan & Natarajan, 2017). Aging, obesity, and a poor diet are common risk factors that create an oxidative environment. This environment can change the body's response to insulin by either increasing resistance to insulin or reducing the ability to tolerate glucose (Hamed et al., 2011). The primary ROS responsible for cellular damage during oxidative stress are superoxide anion radicals (O_2_^−^), hydrogen peroxide (H_2_O_2_), hydroxyl radicals (OH^−^), and singlet oxygen (^1^O_2_) (Nimse & Pal, 2015). These species are volatile and have the potential to harm different cellular constituents, including lipids, proteins, and DNA (Iside et al., 2020).

Nuclear factor erythroid 2–related factor 2 (Nrf2) is a regulatory protein that governs the activation of genes responsible for maintaining electrolyte balance and protecting against oxidative stress (Kovac et al., 2015; Valenzuela et al., 2017). During normal conditions, Keap1 attaches Nrf2 within the cytoplasm. During oxidative stress, Nrf2 is detached from the complex and moves to the nucleus. There, it attaches to the antioxidant response element (ARE), which triggers the activation of gene transcription for NAD(P)H quinone oxidoreductase 1 (NQO1) and heme oxygenase 1 (HO‐1), among other genes (Slocum et al., 2016; Su et al., 2016). Furthermore, under normal circumstances, the body possesses a powerful internal antioxidant system comprising superoxide dismutase (SOD), catalase (CAT), glutathione (GSH), glutathione peroxidase (GPx), and glutathione reductase (GR) that safeguards it against the harmful consequences of excessive generation of ROS (Bhattacharyya et al., 2014; Hong & Lee, 2009). These antioxidants work together to neutralize and eliminate ROS, preventing oxidative damage to cells and tissues.

Oxidative stress‐induced DNA damage results from an inequilibrium between DNA repair and DNA damage (Tao et al., 2021; Pang et al., 2021). This imbalance can accumulate DNA lesions and impair the cell's ability to maintain genomic stability. 8‐oxo‐7,8‐dihydroguanine (8‐oxoG) is a well‐researched DNA oxidation product that is fixed through the base excision repair (BER) pathway initiated by the 8‐oxoG glycosylase1 (BER) pathway (OGG1‐BER) (Wang et al., 2018). The OGG1‐BER pathway is crucial for preserving the integrity of the genome through the repair of DNA damage induced by oxidative stress. Dysregulation of this pathway can result in the accumulation of 8‐oxoG lesions, leading to increased susceptibility to DM complications.

Research has shown that ROS may be a signal molecule to promote cell proliferation and apoptosis (Finkel, 1998). Thus, apoptosis is one of the cellular reactions to oxidative stress and the generation of ROS caused by high glucose (Sun et al., 2012; Xu et al., 2012). Apoptosis is essential for the maintenance of cellular homeostasis (Yu et al., 2023). It serves as a protective mechanism to eliminate damaged or dysfunctional cells, preventing the spread of potential harm throughout the organism. Apoptosis is controlled by a balance involving pro‐ and anti‐apoptotic proteins. The BCL2 family comprises pro‐apoptotic proteins (Bax and Bak) and anti‐apoptotic proteins (Bcl‐2 and Bcl‐x). These proteins cooperate to determine whether cells undergo apoptosis or survival (Hagenbuchner et al., 2012). This process is essential for maintaining cellular homeostasis and inhibiting disease development. Bcl‐2 is an anti‐apoptotic protein that prevents apoptosis by inhibiting Bax/Bak oligomerization, which enhances mitochondrial membrane permeability and inhibits Cyto‐C release (Ren et al., 2020). Previous research has demonstrated reduced Bcl2 expression in response to diabetes stimuli (Ren et al., 2020). Thus, decreased expression of Bcl‐2 may increase apoptosis due to the reduced ability to inhibit Bax/Bak oligomerization following the release of Cyto‐C from the mitochondria. Understanding the control of Bcl‐2 expression in response to diabetic stimuli could offer a valuable understanding of the development and progression of diabetes‐related complications.

Managing hyperglycemia with chemical drugs or insulin causes numerous complications, including insulin‐induced fatty liver (Kandhare et al., 2012; Zhang & Liu, 2011). Therefore, silencing or quenching excess ROS using various natural antioxidants may be a cost‐effective and efficient method for better hyperglycemia management. These natural antioxidants are risk‐free and readily absorbed by cellular systems.

Resveratrol (RES), a natural polyphenol found in various fruits and plants, has undergone thorough investigation due to its numerous health benefits, including its anti‐diabetes, anti‐obesity, anticancer, anti‐inflammatory, antioxidant, and cardioprotective properties, and potential health benefits (Elshaer et al., 2018; Faal et al., 2022). Previous research has demonstrated that RES can effectively mitigate oxidative stress and apoptosis in different cell types (Do et al., 2012; Hoca et al., 2021; Kitada & Koya, 2013; Liu et al., 2014). However, its protective effects against high glucose‐induced damage in HepG2 cells have not been fully elucidated. Hence, our study seeks to fill this knowledge gap and present valuable perceptions of the therapeutic potential of RES in managing oxidative stress and apoptosis. In addition, the findings may aid in creating novel strategies for preventing or treating diabetic complications.

## 2 MATERIALS AND METHODS

### 2.1 Materials

The consumables and reagents for tissue culture were acquired from Sigma–Aldrich (St. Louis, MO, USA). HepG2 cells were generously provided by Prof JL Marnewick from the Cape Peninsula University of Technology, South Africa. D‐glucose and resveratrol were acquired from Sigma‐Aldrich. The quantitative polymerase chain reaction (qPCR) consumables and reagents were acquired from Bio‐Rad (Hercules, CA, USA), while the primer sequences were manufactured by Inqaba Biotechnical Industries (Pretoria, South Africa).

## 3 METHODS

### 3.1 Study design

The glucose and resveratrol treatment concentrations were selected based on the literature. Previous studies established hyperglycemia at 50 and 40 mM (Chandrasekaran et al., 2010; Chu et al., 2011; Kapoor & Kakkar, 2012; Leinninger et al., 2004; Varma et al., 2005). In this study, we used 40 mM to establish hyperglycemia. The resveratrol (RES) concentrations were determined using previous studies (Cheng et al., 2012; Khan et al., 2013; Poonprasartporn & Chan, 2022). This study used 25 and 50 μM concentrations of RES prepared in 100% dimethyl sulfoxide (DMSO). Cells were categorized into six groups: control groups were cultured in normal complete culture medium (CCM), low resveratrol (LR; cultured in normal CCM with 25 μM RES), high resveratrol (HR; cultured in normal CCM with 50 μM RES), high glucose (HG; cultured in normal CCM with 40 mM glucose), LR + HG (cultured in normal CCM with 25 μM RES and 40 mM glucose), and HR + HG (cultured in normal CCM with 50 μM RES and 40 mM glucose).

### 3.2 Cell culture

The human hepatoma G2 (HepG2) cell line is derived from human hepatoma tissue. Many studies have used the HepG2 cell line to study hyperglycemia in vitro (Chandrasekaran et al., 2010; Shokrzadeh et al., 2016; Zhou et al., 2021). Moreover, they are reliable, easy to culture, and well characterized. Our study used the HepG2 cell line to establish hyperglycemia. HepG2 cells were cultured in monolayers (10^6^ cells per flask) in 25 cm^3^ flasks containing Eagle's minimum essential medium (EMEM) supplemented with 10% fetal bovine serum (FBS), 1% pen/strep/fungizone (PSF), and 1% L‐glutamine. Cells were cultured in a controlled environment with high humidity and a temperature of 37°C, supplemented with 5% CO_2_. The cells were rinsed with phosphate‐buffered saline (PBS) containing a 0.1 M concentration of phosphate. Once the cells reached a confluence of 70%–80%, they were exposed to RES (25 and 50 μM) and HG (40 mM) and left to incubate for 48 and 72 h (h). The cells were then removed using trypsin and counted using the trypan blue exclusion technique. The cell suspensions were diluted at a ratio of 1:5 using 60 μL of CCM, 20 μL of cell suspension, and 20 μL of trypan blue solution. The mixture was then incubated for 5 min at room temperature (RT). Then, a 22 × 22 cm coverslip was placed on a clean hemocytometer, and 10 μL of a well‐mixed counting solution was dispensed into the hemocytometer. Using a microscope, the number of living cells was determined using the standard equation (live cell average × 5 (dilution factor) × 10,000 = cells/mL).

### 3.3 RNA isolation and cDNA synthesis

The isolation of total RNA was performed using a QIAzol extraction reagent (Qiagen, Hilden, Germany) and a previously published protocol based on the manufacturer's instructions. Briefly, a 1:1 ratio of Trizol and PBS was introduced into the flask and, subsequently, incubated at RT for a duration of 2 min. Subsequently, cells were detached from the flask's surface using a cell scraper, moved into a microcentrifuge tube, and subjected to a freezing temperature of −80°C. Next, the samples were defrosted, and 100 μL of chloroform was introduced to each tube. The tubes were vigorously agitated for 15 s, incubated for 2–3 min at RT, and centrifuged (15 min, 4°C, 12000 ***g***). Thereafter, the aqueous phase was transferred to a new tube, 250 μL of isopropanol was added, and the tubes were mixed and incubated overnight at −80°C. Next, samples were defrosted and centrifuged (4°C, 12000 ***g***, 20 min), and the pellets were retained. Pellets were washed using 500 μL of cold 75% ethanol. The tubes were then flicked to loosen the pellet and centrifuged at 4°C, 7400 ***g*** for 15 min. Ethanol was removed using a pipette without agitating the pellet. The samples were allowed to dry for a duration of 1.5 h. The pellet was then resuspended in 15 μL of nuclease‐free water and incubated for 2–3 min at RT. Thereafter, the Nanodrop system (Nanodrop Technologies, Wilmington, USA). The integrity was evaluated based on the A260/A280 ratio used to evaluate RNA integrity. The iScript cDNA synthesis kit (Bio‐Rad) was used to perform cDNA synthesis according to the manufacturer's instructions. The reaction mixture consisted of 4 μL 5× iScript reaction mix, 1 μL iScript reverse transcriptase, 14 μL nuclease‐free water, and 1 μL of each RNA sample. After cDNA synthesis, 80 μL of nuclease‐free water was added to each tube, and the samples were stored at −20°C until they were needed for qPCR.

### 3.4 Quantitative polymerase chain reaction (qPCR)

qPCR was used to evaluate gene expression. A reaction mixture of 5 μL SsoAdvanced™ Universal SYBR® Green Supermix (Bio‐Rad), 1.5 μL cDNA, 0.5 μL forward and reverse primers, and 2.5 μL nuclease‐free water was made up to 10 μL. All primers were acquired from Inqaba Biotechnical Industries (Pty) Ltd. The mRNA expression of *Gpx1* (Forward 5′ AAGGTGCTGCTCATTGAGAATG 3′; reverse 5′ CGTCTGGACCTACCAGGAACTT 3′), *CAT* (forward 5′ ACGAGATGGCACACTTTGACAG 3′; reverse 5′ TGGGTTTCTCTTCTGGCTATGG 3′), *SOD* (forward 5′ AGGATTAACTGAAGGCGAGCAT 3′; reverse 5′ TCTACAGTTAGCAGGCCAGCAG 3′), *Nrf2* (forward 5′ AGTGGATCTGCCAACTACTC 3′; reverse 5′ CATCTACAAACGGGAATGTCTG 3′), *BCL‐2* (forward 5′ TGTGGAGAGCGTCAACCGGGAG 3′; reverse 5′ ATCAAACAGAGGCCGCATGCTG 3′), *NQO1* (forward 5′ GAAGAGCACTGATCGTACTGGC 3′; reverse 5′ GGATACTGAAAGTTCGCAGGG 3′), and *OGG1* (forward 5′ GCATCGTACTCTAAGCCTCCAC 3′; reverse 5′ AGGACTTTGCTCCCTCCAC 3′) were investigated. GAPDH (forward 5′ TCCACCACCCTGTTGCTGTA 3′; reverse 5′ ACCACAGTCCATGCCATCAC 3′) was utilized in this assay as a house‐keeping gene, with three replicates per treatment. The initial denaturation occurred at 95°C (2 min), followed by 40 cycles of denaturation (95°C; 15 s), annealing (40 s; *CAT*, *S0D*, *GPx*, *OGG1*—60°C; *Nrf2*, *NQO1*, Bcl‐2—55°C), and extension (72°C; 30 s). The Livak and Schmittgen method was employed to determine changes in relative mRNA expression. In this method, the observed fold change in mRNA expression is represented by $2^{-\Delta \Delta C_{t}}$ (Livak & Schmittgen, 2001).

### 3.5 Lactate dehydrogenase (LDH) assays

The LDH Cytotoxicity Detection Kit (Roche, Mannheim, Germany) evaluated the extracellular LDH release levels. A 96‐well microtiter plate was filled with 100 μL of control and treated cell supernatants in triplicate. The substrate mixture (100 μL) was added to the supernatant and subjected to a 25‐min incubation at room temperature with a catalyst (diaphorase/NAD^+^) and dye solution (INT/sodium lactate). The Elisa microplate reader (Thermo Fisher Scientific; USA) was used to measure the optical density at 490 nm. The findings are presented in the form of mean optical density.

### 3.6 Statistical analysis

All data analyses were performed using GraphPad Prism version 8.0.0 (GraphPad Software, San Diego, CA, USA). The statistical methods employed were one‐way analysis of variance (ANOVA) and the Student's *t*‐test. The experiments were performed in triplicate, and *p* < .05 was deemed statistically significant.

## 4 RESULTS

### 4.1 High glucose decreased the expression of antioxidant enzymes

**FIGURE 1:**
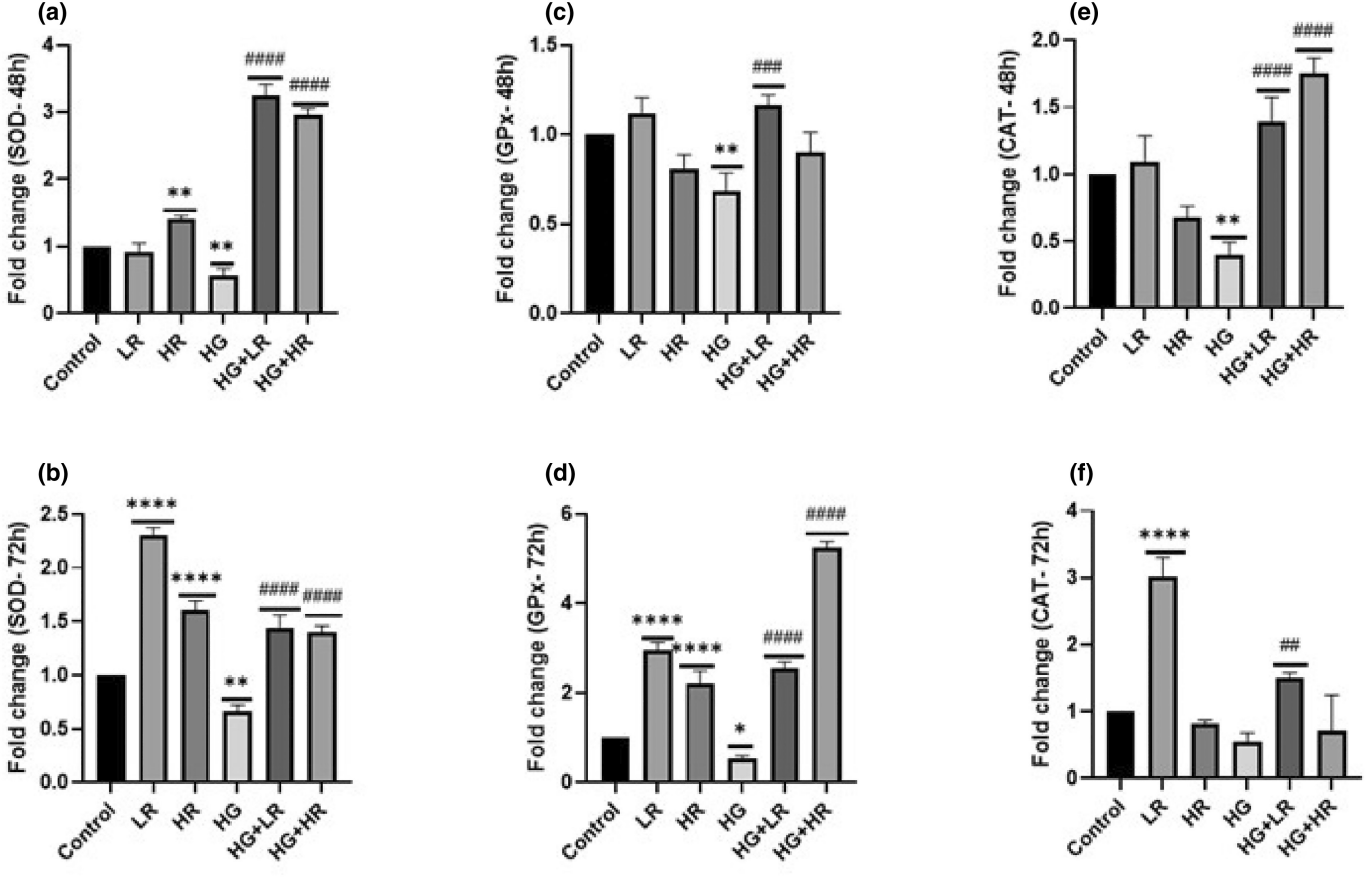
Expression of antioxidant enzyme genes in HepG2 cells treated with high glucose (40 mM) and RES (25 μM and 50 μM) over 48 and 72 h. The mRNA expression was quantified using qPCR. (a) Expression of *SOD* over 48 h. (b) Expression of *SOD* over 72 h. (c) Expression of *GPx* over 48 h. (d) Expression of *GPx1* over 72 h. (e) Expression of *CAT* over 48 h. (f) Expression of *CAT* over 72 h. High glucose significantly decreases all the antioxidant enzymes. Conversely, resveratrol increases the expression levels of all the antioxidant enzymes. GAPDH was used as the housekeeping gene. *p < .05, **p < .01, ***p < .001, ****p < .0001 versus controls and ^#^ *p* < .05, ^##^ *p* < .01, ^###^ *p* < .001, ^####^ *p* < .0001 versus HG. CAT, Catalase; GPx1, glutathione peroxidase 1; HG, High glucose; HR, High resveratrol; LR, Low resveratrol; RES, Resveratrol; SOD, superoxide dismutase.

Oxidative stress results from the excessive buildup of ROS caused by elevated glucose levels within the cells (Poitout and Robertson, 2008). SOD and CAT are the primary antioxidant enzymes responsible for protecting cells from damaging ROS‐mediated effects (Wang & Guo, 2019). Our study evaluated the mRNA expression of *SOD*, *GPx1*, and *CAT* using qPCR. HepG2 cells were exposed to high glucose (HG) (40 mM) for 48 and 72 h. The findings indicated that the expression levels of SOD, GPx1, and CAT were significantly reduced when HepG2 cells were cultured with HG for 48 h (*p* = .0046; *p* = .0045; and *p* = .0011, respectively) (Figure 1a,c,e). When cells were exposed to HG for 72 h, we observed that the expression levels of *SOD* and *GPx1* were significantly decreased (*p* = .0017 and *p* = .043, respectively) (Figure 1b,d), whereas no statistical difference was observed in the expression level of CAT as compared to the normal cells (*p* = .3358; Figure 1f). The results suggest that the effects of HG on antioxidant enzymes are time‐dependent, with longer exposure resulting in greater decreases in *SOD*, *GPx1*, and *CAT* expression levels, indicating that prolonged exposure to HG conditions will gradually reduce the antioxidant potential of cells and allow ROS and RNS to accumulate and damage cellular components. These findings may provide information about oxidative stress‐related cellular damage observed in patients with diabetes.

### 4.2 Resveratrol increased the gene expression of antioxidant enzymes

Since exposure to high glucose reduced the expression of *SOD*, *GPx1*, and *CAT*, we investigated whether RES could counteract the impact of high glucose on these mRNAs. HepG2 cells were cultured with HG + LR and HG + HR over 48 and 72 h (Figure 1). The qPCR findings show that the expression levels of *SOD* increased significantly after exposure to HG + LR and HG + HR over 48 and 72 h (Figure 1a,b) (*p* < .0001) as compared to HG alone. *GPx1* increased significantly after exposure to both HG + LR and HG + HR over 72 h (*p* < .0001) (Figure 1d); however, a significant difference was not observed after exposure to HG + HR over 48 h (*p* = .0525) as compared to HG (Figure 1c). We observed that *CAT* was significantly increased after exposure to both HG + LR and HG + HR over 48 h (*p* < .0001) (Figure 1e) and when exposed to HG + LR for 72 h (*p* = .0069); however, a significant difference was not observed after exposure to HG + HR over 72 h (*p* = .9700) as compared to HG (Figure 1f). These results indicate that RES has the capacity to reverse the negative effects of high glucose on the expression of these antioxidant enzymes.

We investigated the influence of two RES concentrations (25 and 50 μM) on the expression of *SOD*, *GPx1*, and *CAT* (Figure 1) after exposure over 48 and 72 h. The expression levels of *GPx1* and SOD were notably increased after exposure to LR (25 μM) and HR (50 μM) over 72 h as compared to the control (*p* < .0001) (Figure 1b,d,e). No statistical difference was observed in *GPx1* and *CAT* expression levels when HepG2 cells were cultured to LR and HR over 48 h as compared to control (Figure 1b,c). Nonetheless, SOD was notably increased when treated with HR for 48 h (*p* = .0066) (Figure 1a).

### 4.3 The effect of high glucose and resveratrol on the expression of Nrf2 and NQO1 in HepG2 cells

**FIGURE 2:**
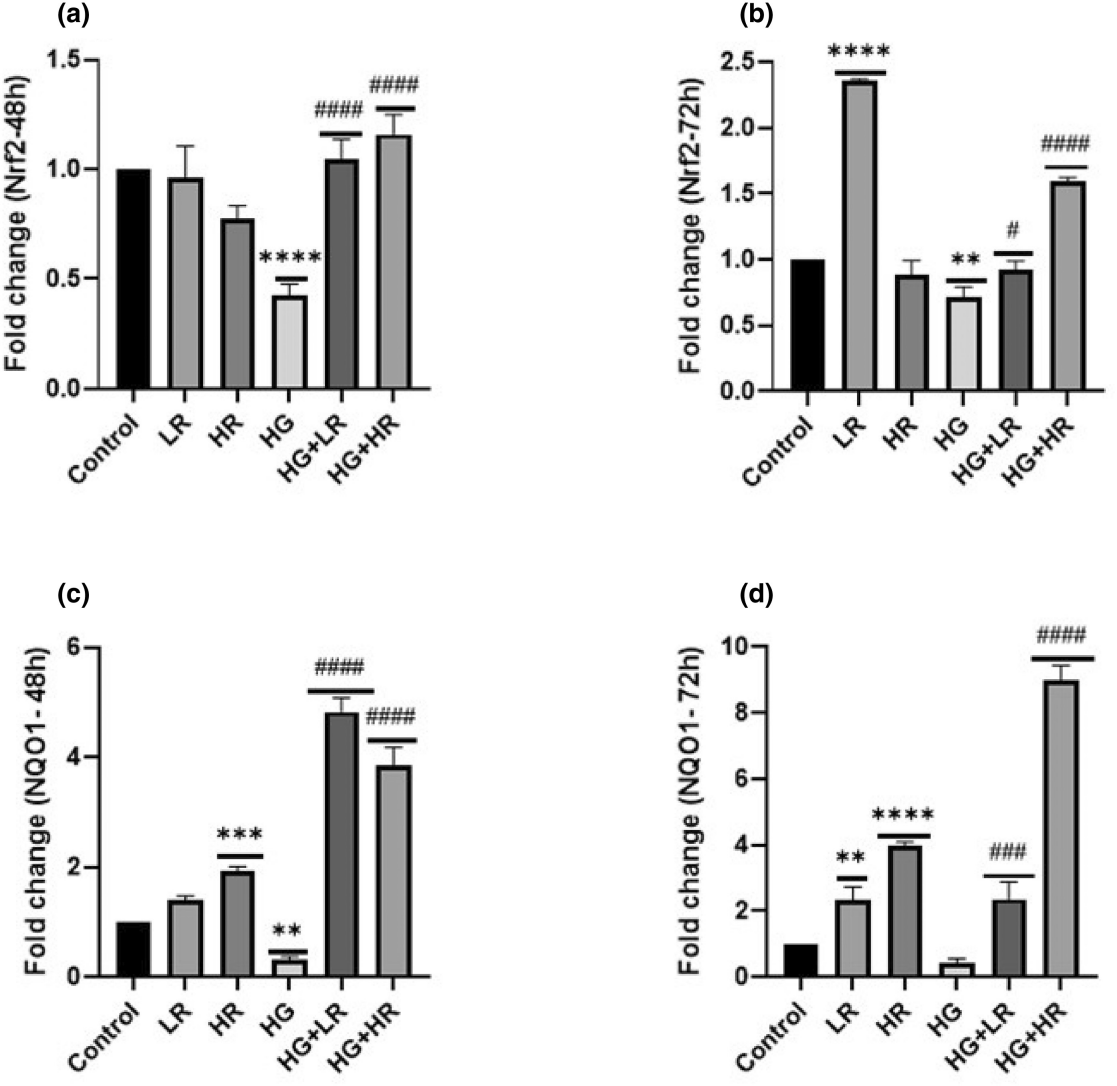
Expression of *Nrf2* and *NQO1* in HepG2 cells treated with high glucose (40 mM) and resveratrol (25 and 50 μM) over 48 and 72 h. High glucose significantly decreased the expression of *Nrf2* and *NQO1*; however, when treated with resveratrol, the expression of *Nrf2* and *NQO1* was significantly increased. *p < .05, **p < .01, ***p < .001, ****p < .0001 versus controls and ^#^ *p* < .05, ^##^ *p* < .01, ^###^ *p* < .001, ^####^ *p* < .0001 versus HG. HG, High glucose; HR, High resveratrol; LR, Low resveratrol; *NQO1*, NAD(P)H quinone oxidoreductase 1; *Nrf2*, Nuclear factor erythroid 2‐related factor 2; RES, Resveratrol.

The mRNA expression of *Nrf2* and *NQO1* was evaluated using qPCR (Figure 2). When treated with HG for 48 and 72 h, the expression of *Nrf2*, a regulator of antioxidant defense, was markedly reduced (*p* < .0001 and *p* = .0010, respectively; Figure 2a,b) relative to the control. *Nrf2* showed no significant difference when treated with LR and HR over 48 h; however, *Nrf2* appeared to increase when treated with LR over 72 h (*p* < .0001; Figure 2a,b) compared to the control. HG + LR and HG + HR significantly increased *Nrf2* expression over 48 h compared to HG group (*p* < .0001). Nrf2 was notably increased when HepG2 cells were cultured with HG + LR and HG + HR for 72 h (*p* = .0125 and *p* < .001, respectively). High glucose significantly decreased the expression of *NQO1* over 48 h (*p* = .0055); however, no statistical difference was observed when treated with HG over 72 h (*p* = .3578). *NQO1* showed no statistical difference when treated with LR over 48 h; however, *NQO1* significantly increased when HepG2 cells were cultured with LR and HR for 72 h compared to the control (*p* = .0042 and *p* < .0001; Figure 2c,d). When treated with HG + LR and HG + HR over 48 and 72 h, the *NQO1* expression level significantly increased compared to HG alone (*p* < .0001).

### 4.4 Effect of high glucose and resveratrol on BCL2 and OGG1 genes

**FIGURE 3:**
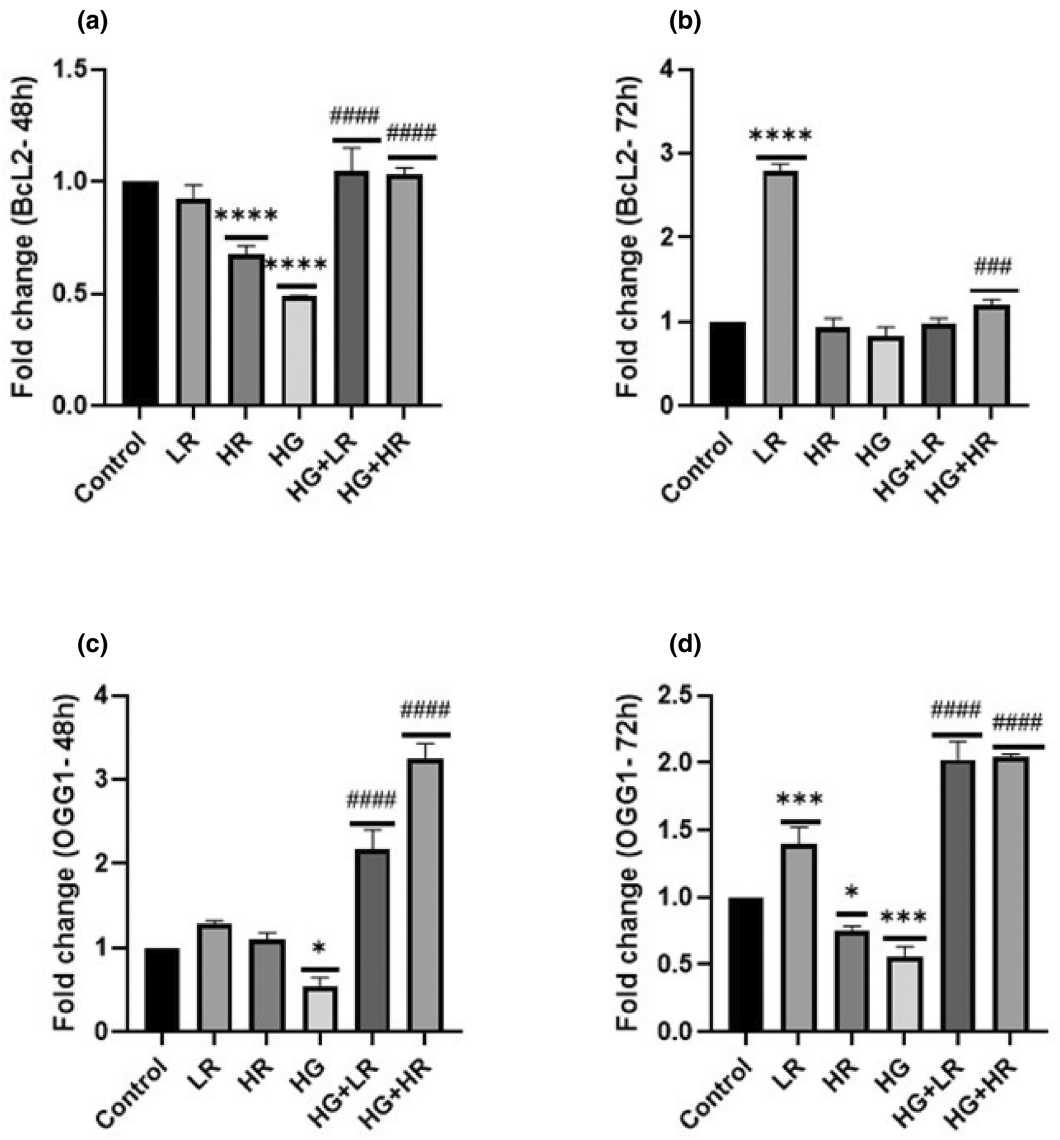
Expression of *Bcl‐2* and *OGG1* in HepG2 cells treated with high glucose (40 mM) and resveratrol (25 and 50 μM) over 48 and 72 h. (a) *Bcl‐2* expression during 48 h exposure. (b) *Bcl‐2* expression during 72 h exposure. (c) *OGG1* expression during 48 h exposure. (d) *OGG1* exposure during 72 h exposure. High glucose reduced the expression of *Bcl‐2* and *OGG1*, whereas resveratrol increased the expression level of *Bcl‐2* and *OGG1*. *p < .05, **p < .01, ***p < .001, ****p < .0001 versus controls and ^#^ *p* < .05, ^##^ *p* < .01, ^###^ *p* < .001, ^####^ *p* < .0001 versus HG. Bcl‐2, B‐cell lymphoma 2; HG, High glucose; HR, High resveratrol; LR, Low resveratrol; OGG1, Oxoguanine glycosylase‐1; RES, Resveratrol.

The expression level of the Bcl‐2 gene related to apoptosis was explored using qPCR. The qPCR findings demonstrated that the expression of Bcl‐2 was significantly reduced when HepG2 cells were cultured with HG over 48 h (*p* < .0001) but not significantly expressed when treated over 72 h (*p* = .1279) as compared to the control (Figure 3). These findings indicated that high glucose levels may have a negative influence on the regulation of Bcl‐2 in HepG2 cells. Bcl‐2 increased when treated with HG + LR and HG + HR over 48 h (*p* < .0001; Figure 3a,b) compared to HG. When treated with HG + HR for 72 h, Bcl‐2 was notably increased (*p* = .005); nonetheless, no statistical difference was observed during exposure to HG + LR over 72 h compared to HG. In addition, no statistical difference was observed in Bcl‐2 when treated with HG + HR over 48 and 72 h as compared to HG. These results indicate that resveratrol could potentially protect HepG2 cells by elevating Bcl‐2 levels.

The involvement of a DNA repair‐related gene was also explored. The qPCR findings demonstrated a significant decrease in the expression of OGG1 when HepG2 cells were cultured with HG over 48 and 72 h (*p* = .0108 and *p* = .0003, respectively; Figure 3c,d). This suggests that exposure to HG may lead to impaired DNA repair mechanisms, specifically affecting the expression of OGG1. The expression of OGG1 was significantly upregulated when HepG2 cells were cultured with HG + LR and G + HR over 48–72 h (*p* < .0001) compared to HG alone. This finding suggests that resveratrol may positively impact OGG1 levels, potentially indicating its role in DNA repair and the oxidative stress response.

### 4.5 LDH activity‐based cytotoxicity assay

**FIGURE 4:**
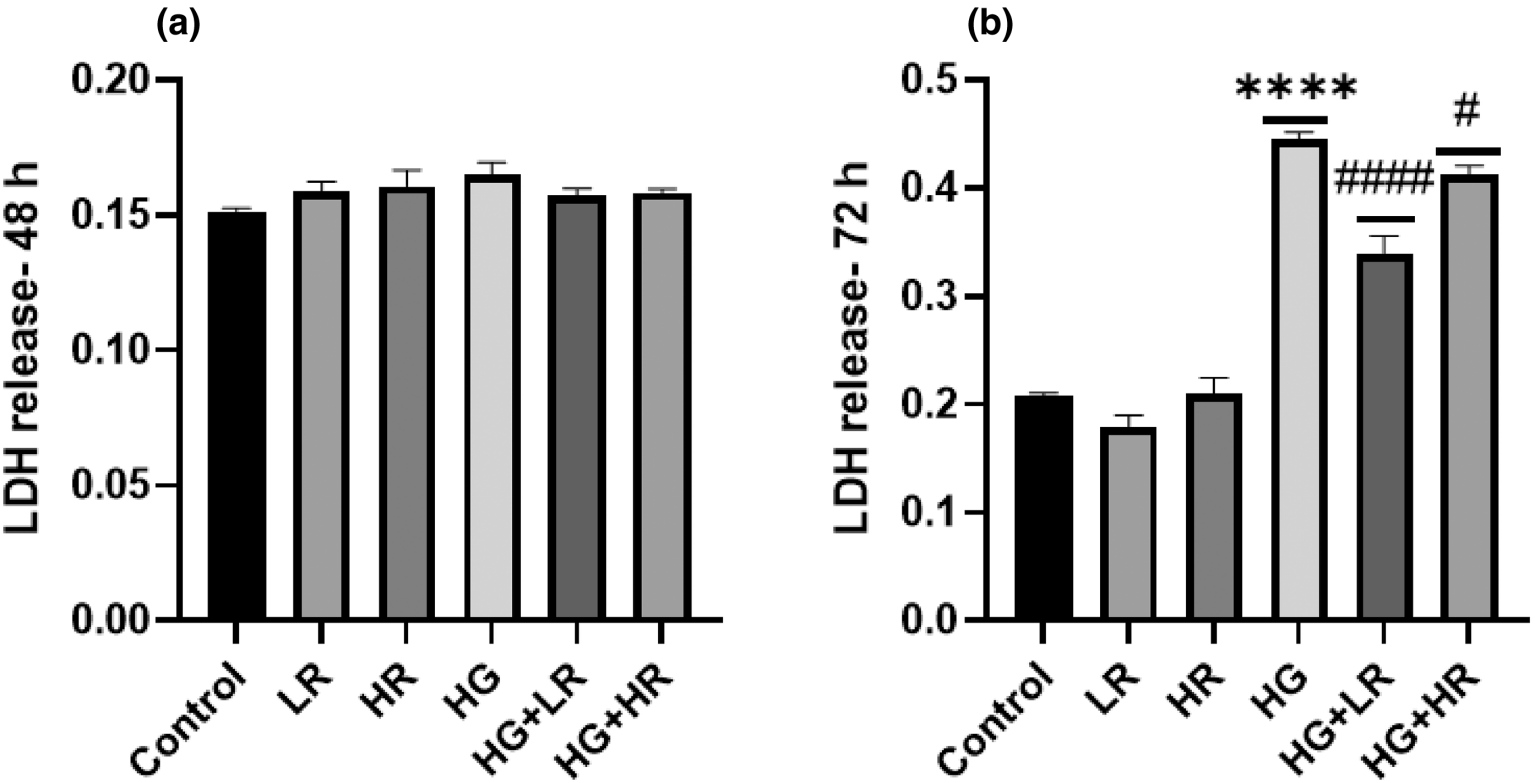
Lactate dehydrogenase release after HepG2 cells were cultured with high glucose (40 mM), Low resveratrol (25 μM), High resveratrol (50 μM), High glucose + Low resveratrol (40 Mm + 25 μM), and High glucose + high resveratrol (40 mM + 50 μM). The results were expressed as the fold change relative to untreated cells. *p < .05, **p < .01, ***p < .001, ****p < .0001 versus controls and ^#^ *p* < .05, ^##^ *p* < .01, ^###^ *p* < .001, ^####^ *p* < .0001 versus HG. HG, High glucose; HR, High resveratrol; LR, Low resveratrol; RES, Resveratrol.

The measurement of LDH release using culture supernatant was employed to determine the integrity of cell membranes. There was no significant difference in the amount of LDH released in HepG2 cells cultured with HG, LR, HR, HG + LR, and HG + HR over 48 h when compared to control (Figure 4a). Additionally, there was no noticeable difference in LDH release when HepG2 cells were cultured with LR and HR over 72 h compared to the control. However, a notable increase was observed when HepG2 cells were cultured with HG over 72 h compared to control cells (*p* < .0001) (Figure 4b). Consequently, our results suggest prolonged exposure to high glucose in HepG2 cells may lead to increased cell damage and death. Interestingly, there was a notable reduction in the release of LDH when HepG2 cells were cultured with HG + LR and HG + HR over 72 h as compared to HG alone (*p* < .0001 and *p* = .0314, respectively) (Figure 4b). These results indicate that RES may provide protection against cell damage and death induced by high glucose in HepG2 cells.

## 5 DISCUSSION

Hyperglycemia‐induced oxidative stress has been demonstrated to play a vital role in the development and progression of diabetes. The present study investigated the expression of oxidative stress and apoptosis‐related genes in HepG2 cells cultured with high glucose and assessed the effect of resveratrol on these genes.

Oxidative stress is the imbalance between oxidant and antioxidant substances (Francisqueti et al., 2017). The overproduction of ROS is linked with the onset of a number of metabolic diseases, including diabetes (Shradha et al., 2010). The current study measured the mRNA expression of CAT, SOD, Gpx1, Nrf2, and NQO1 antioxidants. Our findings showed a significant reduction in the mRNA expression of *SOD*, *CAT*, *GPx1*, *Nrf2*, and *NQO1* in HepG2 cells cultured with high glucose for 48 and 72 h. These results align with previous research demonstrating reduced expression of *SOD*, *CAT*, *GPx*, *Nrf2*, and *NQO1* (Ahmadvand et al., 2023; Subramaniyan & Natarajan, 2017; Wang & Guo, 2019). Antioxidant defenses such as SOD, CAT, and GPx are recognized for their ability to neutralize superoxide anions, lipids, and hydroperoxides in order to protect cells against oxidative stress (Shi et al., 2020). Nrf2 is believed to have a crucial role in regulating the antioxidant defense system due to its ability to bind to the antioxidant response element on active antioxidant enzymes (Shi et al., 2020). Furthermore, NQO1 is implicated in the detoxification of quinones and protecting against oxidative stress. The reduced expression of these antioxidant enzymes and Nrf2 suggests a potential impairment in the cellular defense against oxidative damage caused by high glucose levels.

Resveratrol, a polyphenol phytoalexin, has been studied for its potential therapeutic effects in diabetes (El‐Sayed et al., 2022). Our results demonstrated that treatment with resveratrol reversed the reduction in the mRNA expression of *SOD*, *CAT*, *GPx1*, *Nrf2*, and NQO1 induced by high glucose in HepG2 cells. This suggests that resveratrol has the potential to mitigate oxidative stress caused by high glucose in various diseases. Previous research has also demonstrated increased SOD, CAT, GPx, and Nrf2 expression after resveratrol treatment (Bagul et al., 2012; Hu et al., 2022). These results indicate that resveratrol's capacity to increase the production of antioxidant enzymes and stimulate the Nrf2 pathway may also play a role in its potential therapeutic benefits for diseases associated with oxidative stress. However, further investigations are required on the mechanism underlying resveratrol's effects on antioxidant enzymes and its potential clinical application.

Previous studies have demonstrated that cell death primarily occurs as a result of DNA damage caused by oxidative stress. 8‐oxoG is a well‐researched DNA oxidation that is fixed through a process called base excision repair (BER) initiated by the enzyme OGG1 (Wang et al., 2018). Previous research has demonstrated a reduction in the expression of *OGG1* when exposed to high glucose levels. This finding provides a mechanism for the DNA damage caused by oxidative stress in diabetes (Simone et al., 2008). Moreover, high glucose inhibited OGG1 expression in vivo and in vitro studies (Xie et al., 2020). Consistent with previous research, our findings demonstrated that high glucose reduced the expression of *OGG1*. Intriguingly, *OGG1* mRNA levels increased significantly after treatment with resveratrol, demonstrating enhanced DNA repair. According to our findings, resveratrol may be a potential therapeutic agent for diabetic complications by enhancing DNA repair by upregulating OGG1 expression.

Apoptosis is a programmed cell death triggered by prolonged stress and is strongly controlled by several signaling pathways, comprising the Bcl‐2 family and mitochondrial pathways (Liu et al., 2011). The BCL2 family, including the pro‐ and anti‐apoptotic genes (Bax, Bak, Bcl‐2, and Bcl‐x1), is an important regulator of apoptosis (Rojas‐Rivera et al., 2010). The current study assessed the expression of *Bcl‐2* in HepG2 cells. Our findings revealed that *Bcl‐2* was significantly reduced when HepG2 cells were cultured with high glucose levels. These results align with previous research demonstrating that the expression of *Bcl‐2* was reduced when HepG2 cells were cultured with high glucose (Jiang et al., 2015). Our study further revealed that in HepG2 cells cultured with resveratrol and high glucose, *Bcl‐2* expression was significantly increased. The findings suggest that resveratrol may upregulate the expression of Bcl‐2 to modulate the apoptotic pathway. Additionally, our results revealed that the LDH activity was significantly increased in HepG2 cells over 72 h, whereas in HepG2 cells cultured with high glucose over 48 h, no statistical difference was observed. Previous research also demonstrated increased LDH activity following exposure to high glucose (50 mM) in HepG2 cells (Chandrasekaran et al., 2010). These findings suggest that exposure to HepG2 at a high glucose concentration for prolonged periods, i.e., 72 h, is toxic to HepG2 cells. Surprisingly, HepG2 cells cultured with resveratrol and high glucose significantly decreased the LDH released compared to the high glucose group alone. The results showed that resveratrol has a protective effect against the toxicity caused by high glucose in HepG2 cells. Additional research is required to clarify the underlying mechanism of this protective effect and investigate the potential therapeutic uses of resveratrol in managing high glucose‐induced toxicity in liver cells.

Our research demonstrates that resveratrol displays antioxidant and antiapoptotic properties. This research has certain limitations. This study was conducted in vitro using HepG2 cells and would benefit from further investigations using appropriate animal models, such as diabetes‐induced mice. Moreover, this study only focused on genes implicated in oxidative stress and apoptosis. Future studies should assess functional protein expression in order to determine a correlation with gene expression. Studies in humans, such as clinical trials or dietary supplementation, may provide further insights into resveratrol's antioxidant and antiapoptotic functions. Future research must investigate the potential clinical applications of resveratrol in humans. In addition, it would be intriguing to explore the synergistic influence of resveratrol when combined with other antioxidants or antiapoptotic agents.

## 6 CONCLUSION

Our study demonstrated the potential of resveratrol to mitigate oxidative stress injury and apoptosis in HepG2 cells induced by high glucose levels. The antioxidant stress‐protective effect of resveratrol was observed through its ability to enhance intracellular antioxidants. In the interim, it impeded the process of apoptosis triggered by elevated glucose levels through upregulation of Bcl‐2 mRNA expression. Furthermore, the administration of resveratrol resulted in an upregulation of the DNA repair gene known as OGG1. Hence, it is plausible that RES could be a viable approach to addressing the impairment of the antioxidant response resulting from elevated glucose levels commonly observed in diabetes and metabolic disorders.

## AUTHOR CONTRIBUTIONS

**Abegail Mukhethwa Tshivhase:** Data curation (equal); formal analysis (lead); investigation (equal); methodology (equal); software (equal); validation (equal); visualization (equal); writing – original draft (lead). **Tandi Matsha:** Funding acquisition (lead); resources (lead); supervision (equal); writing – review and editing (supporting). **Shanel Raghubeer:** Conceptualization (lead); formal analysis (supporting); investigation (equal); methodology (lead); project administration (lead); supervision (equal); writing – review and editing (supporting).

## FUNDING INFORMATION

This research project was supported by a grant from the South African Medical Research Council (SAMRC), with funds from National Treasury under its Economic Competitiveness and Support Package (MRC‐RFA‐UFSP‐01‐2013/VMH Study) and South African National Research Foundation (SANRF) (Grant no. 115450).

## CONFLICT OF INTEREST STATEMENT

The authors declare no conflicts of interest.

## ETHICS STATEMENT

This study did not involve animal or human participants.

## Data Availability

The data that support the findings of this study are available on request from the corresponding author.
